# Time poverty and teacher subjective well-being in an accelerated society: a three-wave longitudinal analysis of emotional exhaustion, psychological resilience, and gender

**DOI:** 10.3389/fpsyg.2025.1728219

**Published:** 2025-12-15

**Authors:** Kunyan Wang, Tao Huang, Xinyue Lin, Rong Tan

**Affiliations:** 1 School of Psychology, Northeast Normal University School of Psychology, Changchun, China; 2 School of Preschool Education, Jiangmen Preschool Education College, Jiangmen, China; 3 School of Social Development, East China University of Political Science and Law, Shanghai, China; 4 School of Psychology, Central China Normal University, Wuhan, China

**Keywords:** time poverty, teacher subjective well-being, emotional exhaustion, psychological resilience, gender differences

## Abstract

**Introduction:**

Time poverty, driven by social acceleration, is emerging as a critical threat to teacher subjective well-being (SWB). Yet, the causal mechanisms linking time poverty to diminished SWB and the protective factors that might buffer this detrimental process remain empirically underexplored.

**Methods:**

To address this gap, a semester-long survey was conducted with 645 Chinese teachers. Time poverty and psychological resilience were measured at Time 1 (T1), emotional exhaustion at Time 2 (T2), and subjective well-being at Time 3 (T3). Utilizing this three-wave longitudinal data, our study tested a moderated mediation model to examine whether emotional exhaustion mediates the long-term impact of time poverty on teacher SWB and whether this indirect effect is moderated by psychological resilience and gender.

**Results:**

Results demonstrated that emotional exhaustion was a key mediator in the relationship between time poverty and SWB. Furthermore, the analysis uncovered a dual-path moderation: psychological resilience acted as a buffer, effectively weakening the adverse impact of time poverty on emotional exhaustion. In contrast, gender served as an amplifier, exacerbating the detrimental effect of emotional exhaustion on SWB, with female teachers being significantly more vulnerable than their male counterparts.

**Discussion:**

This study concludes that time poverty profoundly harms teacher SWB, not directly, but by depleting their emotional resources. This finding presents a critical empirical challenge to techno-optimistic narratives, revealing structural time pressure as a significant dark side of digital transformation in education. Our results underscore that for educational administrators, moving beyond fostering individual resilience to systemically building gender-sensitive, “time-friendly” school environments are crucial for combating this structural pressure.

## 1 Introduction

Social acceleration is a core dynamic of modern society, exerting influence across all societal domains (Rosa, 2013). As a key institution for social reproduction, education is invariably impacted by this accelerative trend. In China, this global phenomenon is manifesting with unprecedented scale, driven by the nationwide “Education Digitalization Strategy” and the deep integration of “AI+ Education,” which together are initiating a profound structural transformation of the educational system. Frontline teachers, who are central to this transformation, consequently face significant challenges to their well-being. This pressure is rooted in a structural force of “dynamic stabilization”—a logic wherein constant acceleration in growth and innovation is required merely to maintain the status quo (Rosa and Tang, 2023).

In this context, the integration of “AI+ Education” may function as a double-edged sword. While these innovations promise benefits such as enhanced efficiency and personalized learning (Kamalov et al., 2023), empirical studies indicate they also introduce significant new challenges. The pressure to acquire new technological skills and the intensification of work are increasingly recognized as sources of teacher stress and anxiety (Fernández-Batanero et al., 2021). Furthermore, this digitalization may inadvertently increase teachers’ workloads and exacerbate their work-life conflict (Luo, 2023; Keränen et al., 2025), imposing new burdens and thereby exacerbating challenges to their well-being. Empirical evidence supports this concern; studies indicate high levels of professional burnout and psychological distress among teachers (García-Carmona et al., 2019). Moreover, meta-analyses focusing on Chinese teachers reveal a consistent decline in subjective well-being and a rise in mental health issues since the beginning of the century (Xin et al., 2021; Yu, 2025), a trend that temporally coincides with the intensification of social acceleration and digitalization in education. Worsening professional exhaustion is closely related to higher teacher attrition and lower job satisfaction (Madigan and Kim, 2021), which may pose a threat to the stability of the educational system and the quality of education. Therefore, in an era of technological acceleration, teacher subjective well-being transcends a purely personal, affective matter; it must be regarded as a critical factor determining whether the digital transformation of education culminates in “resonance” or “alienation”.

Teacher subjective well-being is a positive, work-related psychological state derived from the successful fulfillment of professional duties, encompassing the two primary dimensions of school connectedness and teaching self-efficacy (Renshaw et al., 2015). Previous research on its antecedents has primarily followed two paths: one focusing on personal resources, which has confirmed the positive predictive role of traits like mindfulness and emotional intelligence (Chamani et al., 2023; Pan et al., 2022); and another exploring situational factors, which has revealed the positive impact of supportive contexts, such as organizational and collegial support (Skaalvik and Skaalvik, 2018), and the significant negative impact of draining contexts characterized by work overload and role ambiguity (Ao et al., 2023; Macovei et al., 2023).

While existing research has identified multiple variables (job demands/resources, personal demands/resources) influencing teacher subjective well-being (Li et al., 2025), it has paid insufficient attention to a core issue stemming from social acceleration: time alienation. Current approaches to time overload have primarily defined it using objective standards, such as actual work hours or the quantity of available discretionary time (e.g., Gershuny, 2011; Kalenkoski et al., 2011). For example, Li and Du (2023) have defined time overload using a threshold of 1.25 times the median social labor time. However, modern society is characterized by multitasking, and high-intensity task processing models (Rudd, 2019), meaning individuals may feel exhausted even when their work hours are not objectively overloaded. In this context, the subjective experience of time poverty more accurately reflects an individual’s perception of time availability. This distinction is critical, as emerging research suggests that subjective time poverty may have a stronger impact on individual well-being than objectively defined time scarcity (Dong and Sun, 2025). Therefore, it is necessary to focus on subjective time poverty. A central paradox of social acceleration theory is that technological advancements, intended to save time, often intensify the sense of time scarcity (Rosa, 2013). In the educational context, the proliferation of digital tools has created “always-on” multitasking pressures (Keränen et al., 2025), while the integration of AI has further intensified demands for skill iteration and self-optimization (Luo, 2023). Together, these factors may constitute the temporal acceleration experienced by teachers. Consequently, the subjective experience of insufficient time availability, known as time poverty, and its relationship with teacher subjective well-being, represents a critical and underexplored area of research.

Teacher time poverty is defined as the subjective perception of having insufficient time to complete one’s professional tasks and obligations (Liu et al., 2023). COR theory offers a robust framework for understanding its effects, positing that psychological distress stems from the actual or threatened loss of valued resources (Hobfoll et al., 2018). Time is a foundational resource, essential for acquiring other resources (e.g., income via work, social support via engagement) and for overall well-being (Holmes, 2023; Rosa and Pu, 2022). From a COR perspective, time poverty represents a significant resource loss that can trigger feelings of insecurity and threat, thereby depleting an individual’s resource pool and diminishing subjective well-being.

This theoretical link is supported by empirical evidence. Studies have consistently found a significant negative correlation between time poverty and subjective well-being (Batur et al., 2019; Zhu et al., 2025). Furthermore, time poverty significantly reduces life satisfaction (Liu et al., 2023), a key component of subjective well-being. Despite this established negative correlation, the causal direction of the relationship remains unclear. The cross-sectional design of most prior research makes it impossible to determine whether time poverty leads to decreased well-being or if lower well-being exacerbates the perception of time poverty. To clarify the causal precedence of time poverty as a persistent stressor, the present study employs a three-wave longitudinal design to test its direct predictive effect on subjective well-being. Based on the preceding evidence, we propose hypothesis 1: Time poverty will negatively predict teacher subjective well-being.

### 1.1 The mediating role of emotional exhaustion

To elucidate the mechanism through which time poverty affects teacher subjective well-being, this study proposes emotional exhaustion as a key mediating variable. Emotional exhaustion is a state of affective depletion characterized by feeling emotionally overextended and drained of one’s psychological resources (Maslach, 2003). COR theory provides the theoretical framework for this mediation, particularly through its concept of “loss spirals”. The theory posits that an initial resource loss compels individuals to invest remaining resources to compensate, which can trigger a cascade of further depletion, eroding their overall resource pool (Hobfoll et al., 2018).

Within this framework, time poverty constitutes an initial loss of a crucial “condition resource”. To cope with this deficit, teachers must invest their finite “energy resources,” such as sustained emotional and cognitive effort. This over-investment can lead to the depletion of these reserves, a state which defines emotional exhaustion—itself a secondary resource loss. This state of resource depletion, in turn, has cascading negative effects on subjective well-being. According to the loss spiral principle, teachers experiencing emotional exhaustion have a diminished capacity to protect existing resources or acquire new ones. They may lack the psychological energy to maintain positive relationships with colleagues and students (impairing school connectedness) or to derive a sense of accomplishment from their work (impairing teaching self-efficacy). Existing research supports both pathways of this proposed model. First, studies have confirmed that time poverty is a significant predictor of emotional exhaustion (Liu and Wang, 2024; Zhu et al., 2025). Second, emotional exhaustion is well-established as a potent negative predictor of subjective well-being across diverse populations (Cui, 2022; Lee et al., 2020; Wang et al., 2020). Based on this theoretical reasoning and empirical evidence, we propose hypothesis 2: Emotional exhaustion mediates the relationship between teacher time poverty and subjective well-being.

### 1.2 The moderated role of resilience

Psychological resilience, the capacity for positive adaptation in the context of significant adversity (Campbell-Sills and Stein, 2007), is a critical personal resource. Drawing upon both COR theory and the Stress-Buffering Model, we propose that resilience functions as a moderator in the process linking time poverty to subjective well-being. Specifically, we theorize that resilience plays a dual role along this pathway: first as a “buffer” and second as a “restorer”.

First, resilience may act as a buffer on the path from time poverty to emotional exhaustion. According to the Stress-Buffering Model, personal resources can attenuate the detrimental relationship between stressors and negative health outcomes (Cohen and Wills, 1985). As a key personal resource, resilience may therefore buffer the impact of time poverty (the stressor) on emotional exhaustion (the outcome). This proposition is consistent with existing research; for instance, resilience has been shown to buffer the effect of role overload on sleep disturbance (Irani et al., 2023) and to moderate the relationship between sleep disturbance and teacher burnout (Yang et al., 2023). However, this buffering role has rarely been examined in the context of time poverty as a specific stressor.

Second, resilience may function as a restorer on the path from emotional exhaustion to subjective well-being. According to the “loss spiral” tenet of COR theory, a significant resource loss like emotional exhaustion renders individuals more vulnerable to subsequent, broader resource losses. Resilience may help to interrupt this loss spiral. Highly resilient teachers likely possess a greater capacity for recovery, are more adept at finding meaning and mobilizing support, and are thus better able to replenish their resources. This capacity would protect their remaining resource pool and help maintain their subjective well-being. Indeed, research confirms that resilience is a potent negative predictor of burnout and exerts a direct protective effect on teacher well-being (Li, 2023; Pozo-Rico et al., 2023).

Based on this framework, we propose:

*Hypothesis 3a:* Resilience moderates the relationship between time poverty and emotional exhaustion.

*Hypothesis 3b:* Resilience moderates the relationship between emotional exhaustion and subjective well-being.

### 1.3 The moderated role of gender

Beyond individual resources, gender, as a significant sociocultural factor, may also moderate the processes linking time poverty to teacher well-being. According to Social Role Theory, societal expectations shape differences between men and women in behavioral patterns, role responsibilities, and stress experiences (Eagly and Wood, 1999). Drawing on this perspective, we propose that gender moderates both stages of the mediation chain.

First, on the path from time poverty to emotional exhaustion, female teachers may be more vulnerable. Societal norms often expect women to shoulder a greater share of domestic and caregiving responsibilities (i.e., “non-work labor”) (Zilanawala, 2016). Consequently, time poverty is more likely to induce work–family conflict for female teachers, thereby amplifying the impact of this resource loss and leading to more severe emotional exhaustion. Second, gender may moderate the link between emotional exhaustion and subjective well-being. Women are often socialized into roles emphasizing caregiving and emotional maintenance (Eagly, 1987). As a result, when experiencing emotional exhaustion, female teachers may perceive it not just as resource depletion, but also as a failure to fulfill a core social role. This additional layer of negative self-appraisal could intensify the detrimental effect of exhaustion on their subjective well-being.

Existing research indicates that women generally report higher levels of time poverty (Qi and Dong, 2018; Rodgers, 2023), and gender has been identified as a moderator in various pathways to well-being (Nasir Abbasi et al., 2024; Zhang et al., 2023). However, whether gender moderates the relationships among time poverty, emotional exhaustion, and subjective well-being specifically within the teacher population remains unexplored. To address this gap, we propose:

*Hypothesis 4a:* Gender moderates the relationship between time poverty and emotional exhaustion.

*Hypothesis 4b:* Gender moderates the relationship between emotional exhaustion and subjective well-being.

### 1.4 The present study

**Figure 1:**
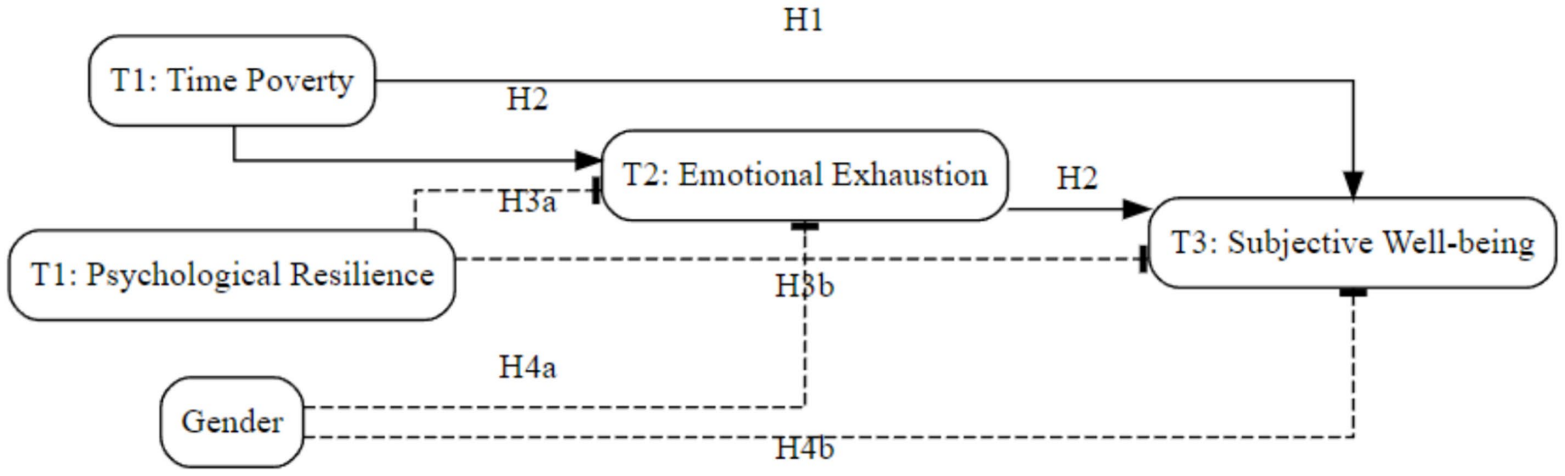
Conceptual model.

Teacher time poverty has become a pervasive issue (Liu et al., 2023). In China, this challenge is further amplified as the country is comprehensively promoting its educational digital transformation with unprecedented intensity. National policy also explicitly states the need to advance the digital construction of the teacher workforce, urging teachers to actively adapt to new technological changes and integrate them with teaching and learning (Ministry of Education, 2023). This top-down mandate is likely to create unique pressures and exacerbate the challenge of time poverty for teachers. As one of the world’s largest teacher workforces, the Chinese sample is highly representative and provides a critical case, offering valuable insights for other developing countries undergoing similar, large-scale digital transformations. To address the challenges to teacher well-being posed by the digital transformation of Chinese education in an era of social acceleration, the present study investigates the impact of teacher time poverty on subjective well-being. Our primary objective is to elucidate the underlying psychological mechanisms and key boundary conditions of this relationship. Drawing upon Conservation of Resources theory, the Stress-Buffering Model, and Social Role Theory, we propose and test a moderated mediation model (see Figure 1). In this model, emotional exhaustion is positioned as a mediator of the relationship between time poverty and subjective well-being. Furthermore, we examine psychological resilience and gender as moderators at distinct stages of this mediation pathway. The model will be tested using a three-wave longitudinal design with two-month intervals. We anticipate that this research will not only advance theoretical understanding of the psychological and social factors affecting teacher well-being in a rapidly changing environment but will also provide an empirical basis for developing effective support strategies to ensure the sustainable success of educational digitalization initiatives.

## 2 Materials and methods

### 2.1 Participants and procedure

The participants were in-service teachers from primary and secondary schools in the Jiangxi and Fujian provinces of China. The study protocol followed the ethical guidelines of the Declaration of Helsinki and received approval from the Institutional Review Board at the authors’ university.

A three-wave longitudinal design was employed, with data collected via WJX, an online survey platform. Data were gathered at three time points within a single academic year (February 2025, April 2025, and July 2025), corresponding to the beginning, middle, and end of the semester. The two-month intervals were chosen to capture the dynamics of resource depletion and well-being, a process that typically unfolds over several months (Salanova et al., 2006).

Prior to the first survey, all participants were fully informed of the study’s purpose, the voluntary nature of their participation, and data confidentiality procedures. Electronic informed consent was obtained from all participants. At Time 1, we measured time poverty, psychological resilience, and demographic variables. At Time 2, we measured emotional exhaustion. At Time 3, we measured teacher subjective well-being.

The initial sample at T1 consisted of 1,066 teachers. Of these, 705 completed the T2 survey, and 645 completed all three waves, resulting in a final retention rate of 60.5%. The final sample of 645 teachers included 198 males (30.7%) and 447 females (69.3%). Regarding teaching experience, 53.3% (*n* = 344) had 0–5 years, 30.4% (*n* = 196) had 6–15 years, and 16.3% (*n* = 105) had more than 15 years. Detailed demographic information is presented in Table 1.

**Table 1:** Participants’ demographic information.

Demographic information	Category	Frequency	Percent (%)
Gender	Male	199	30.7%
	Female	447	69.3%
Length of teaching	0 ~ 5 years	344	53.3%
	6 ~ 15 years	196	30.4%
	>15 years	105	16.3%
Educational background	Associate’s degree	31	6.3%
	Bachelor’s degree	369	75.2%
	Master’s degree	91	18.5%
Professional title	Primary	399	61.9%
	Middle	199	29.6%
	Senior	55	8.5%

### 2.2 Attrition analysis

To examine potential attrition bias, we compared the retained group (*n* = 645) with the attrition group (*n* = 421) on key demographic variables. A series of chi-square tests revealed no significant differences in gender (*χ^2^* = 1.08, *p* = 0.299), teaching experience (*χ^2^* = 4.12, *p* = 0.127), educational background (*χ^2^* = 4.38, *p* = 0.223), or professional title (*χ^2^* = 1.34, *p* = 0.512). These results suggest that sample attrition was largely random and is unlikely to have systematically biased the findings.

### 2.3 Measures

#### 2.3.1 Teachers’ time poverty scale

Time poverty was assessed by the Teachers’ Time Poverty Scale (Liu et al., 2023). This questionnaire comprises 7 items (e.g., “There is no autonomy in the allocation of my time”), with each item rated on a 5-point Likert scale (“1” indicating strongly disagree, and “5” indicating strongly agree). The scale has a single-factor structure. Higher scores on the questionnaire correspond to higher levels of perception of time poverty. The scale has demonstrated suitability for use with Chinese teachers (Zhu et al., 2025) and exhibited good reliability in the current study (Cronbach’s *α* = 0.88) at T1.

#### 2.3.2 Emotional exhaustion scale

The Emotional Exhaustion Scale of the Model Burnout Inventory for Educators (MBI-ES), developed specifically for educators, was adopted by Maslach and Jackson (1981). The scale consists of nine items (e.g., “I feel exhausted when I leave work”). A 5-point scale (1 = strongly disagree, 2 = disagree, 3 = fairly agree, 4 = agree, 5 = strongly agree) is used, with higher scores indicating greater emotional exhaustion. In the current study, the Emotional Exhaustion Scale demonstrated strong internal consistency, with a Cronbach’s *α* of 0.93 at T2.

#### 2.3.3 Connor-Davidson resilience scale

The 10-item Connor-Davidson Resilience Scale was employed to measure psychological resilience. This unidimensional questionnaire was developed by Campbell-Sills and Stein (2007) and contains 10 items (e.g., “I am able to adapt to change”). Respondents rated each item on a 5-point Likert scale ranging from 0 (never) to 4 (always), with higher scores indicating greater resilience. The scale has shown sound reliability and validity among teacher samples (Lu et al., 2024). In the present study, the scale also demonstrated good internal consistency (Cronbach’s α = 0.92) at T1.

#### 2.3.4 Teacher subjective well-being questionnaire

The assessment of subjective well-being employed the revised Teacher Subjective Well-being Questionnaire (Renshaw et al., 2015), as validated by Xie et al. (2024). This scale contains 8 items, including two dimensions: Sense of school connectedness (4 items, e.g., “I really feel comfortable at this school”), sense of teaching efficacy (4 items, e.g., “I think my teaching methods are effective and useful”) and used a Likert 4-point scoring method, ranging from 1 (almost never) to 4 (almost always). The higher total score on this scale indicates higher degrees of teacher’s subjective well-being. In this study, the Cronbach’s α of total questionnaire was 0.889, for sense of school connectedness and sense of teaching efficacy, it was 0.83 and 0.84, respectively at T3.

### 2.4 Statistical analyses

Data analysis was performed using SPSS 25.0 and R (Version 4.5.1). The analysis followed three steps. First, Harman’s single-factor test was utilized to examine potential common method bias. Second, descriptive statistics and Pearson correlation analyses were conducted for all primary variables using SPSS. Finally, Structural Equation Modeling (SEM) was employed to test the hypothesized moderated mediation model using the lavaan package in R (Rosseel, 2012). We constructed a path model to examine the mediating role of emotional exhaustion and the moderating roles of psychological resilience and gender. Model fit was evaluated using the Chi-square statistic (*χ*^2^), Comparative Fit Index (CFI), Tucker-Lewis Index (TLI), Root Mean Square Error of Approximation (RMSEA), and Standardized Root Mean Square Residual (SRMR). To test the significance of indirect and conditional effects, we used bias-corrected bootstrapping with 1,000 resamples to generate 95% confidence intervals (CI). An effect was considered statistically significant if the 95% CI did not contain zero (Baraff et al., 2016).

## 3 Results

### 3.1 Common method bias

We used Harman’s single-factor analysis to test possible common method bias in our study (Tang and Wen, 2020). It was found that there were 6 factors with eigenvalues above 1 and that the variance explained by the first common factor was 28.87%, which was below the empirical criterion of 40% (Podsakoff et al., 2003). Therefore, there are no serious common method biases in this study.

### 3.2 Descriptive statistics and correlation analysis of main variables

Descriptive statistics (means and standard deviations) and correlations for the main study variables are presented in Table 2. Specifically, resilience and teacher’s subjective well-being were significantly and negatively associated with time poverty (*r* = −0.009, *p* < 0.05; *r* = −0.261, *p* < 0.01, respectively), while emotional exhaustion was significantly positively correlated with time poverty (*r* = 0.716, *p* < 0.01). Furthermore, emotional exhaustion was significantly negatively correlated with resilience and teacher’s subjective well-being (*r* = −0.263, *p* < 0.05; *r* = −0.384, *p* < 0.01, respectively). Resilience is significantly positively correlated with teacher subjective well-being (*r* = 0.619, *p* < 0.01).

**Table 2:** Results of descriptive statistics and correlation analysis.

Variables	*M*	*SD*	1	2	3	4
1. TP (T1)	24.581	5.542	1			
2. EE (T2)	28.606	7.324	0.716^**^	1		
3. Resilience (T1)	33.831	6.198	−0.099^*^	−0.263^**^	1	
4. SWB (T3)	18.831	4.486	−0.261^**^	−0.384^**^	0.471^**^	1

*N* = 645; M = mean, SD = standard deviation; TP = Time Poverty; EE = Emotional Exhaustion; SWB = Subjective Well-being; ^*^p < 0.05, ^**^p < 0.01, ^***^p < 0.001, the same as below.

### 3.3 Testing the measurement model

Prior to testing the structural hypotheses, a Confirmatory Factor Analysis was conducted to assess the construct validity and measurement model fit. The measurement model consisted of four latent variables: Time Poverty, Emotional Exhaustion, Psychological Resilience, and Subjective Well-being. The results indicated that the measurement model demonstrated a satisfactory fit to the data: *χ*^2^ = 1347.373, df = 487, *χ*^2^/df = 2.767; CFI = 0.935; TLI = 0.929; RMSEA = 0.052; SRMR = 0.032. All factor loadings were significant, supporting the distinctiveness of the study constructs.

### 3.4 Testing the structural model and direct/indirect effects

We employed Structural Equation Modeling using the lavaan package in R to test the hypothesized moderated mediation model. The structural model exhibited a good fit to the data: *χ*^2^ = 1368.671, df = 518, *χ*^2^/df = 2.642; CFI = 0.935; TLI = 0.930; RMSEA = 0.050; SRMR = 0.031.

The path analysis results (standardized coefficients, *β*) are presented in Table 3. Regarding the direct effects, Time Poverty at T1 was a significant positive predictor of Emotional Exhaustion at T2 (*β* = 0.382, *p* < 0.001). Furthermore, Emotional Exhaustion at T2 significantly and negatively predicted Subjective Well-being at T3 (*β* = −0.380, *p* < 0.001). Notably, the direct effect of Time Poverty on Subjective Well-being remained significant in the SEM model (*β* = −0.115, *p* = 0.017), supporting Hypothesis 1.

**Table 3:** Path coefficients and structural model results.

Path	*β*	*S.E.*	z-value	*p*
Direct effects				
TP → EE	0.382	0.053	8.193	< 0.001
Resilience → EE	−0.159	0.049	−3.682	< 0.001
Sex → EE	0.038	0.090	1.039	0.299
TP → SWB	−0.115	0.060	−2.390	0.017
EE → SWB	−0.380	0.053	−7.914	< 0.001
Resilience → SWB	0.256	0.058	5.474	< 0.001
Sex → SWB	0.047	0.100	1.261	0.207

*N* = 645. Model Fit: χ^2^ = 1368.671, df = 518, *p* < 0.001; χ^2^/df = 2.642; CFI = 0.935; TLI = 0.930; RMSEA = 0.050; SRMR = 0.031. Sex: 0 = Female, 1 = Male; SWB = Subjective Well-being; EE = Emotional Exhaustion; TP = Time Poverty.

To test the mediation hypothesis 2, we examined the indirect effect of Time Poverty on Subjective Well-being via Emotional Exhaustion using bias-corrected bootstrapping with 1,000 resamples. The results confirmed a significant negative indirect effect (Estimate = −0.145, *p* < 0.001) (see Table 4). Given that the direct effect of Time Poverty on Subjective Well-being was also significant, the results indicate that Emotional Exhaustion plays a partial mediating role in this relationship, supporting Hypothesis 2.

**Table 4:** Decomposition of direct, indirect, and total effects.

Effect path	*β*	*S. E.*	z-value	*p*
Total effect				
TP → SWB	−0.260	0.060	−5.364	< 0.001
Direct effect				
TP → SWB	−0.115	0.060	−2.390	0.017
Indirect effect (mediation)				
TP → EE → SWB	−0.145	0.031	−5.902	< 0.001

TP = Time Poverty; SWB = Subjective Well-being; EE = Emotional Exhaustion.

### 3.5 Testing the moderating effects

We further examined the moderating roles of Psychological Resilience and Gender (Male = 1, Female = 0). The interaction terms were created using mean-centered continuous variables.

**Figure 2:**
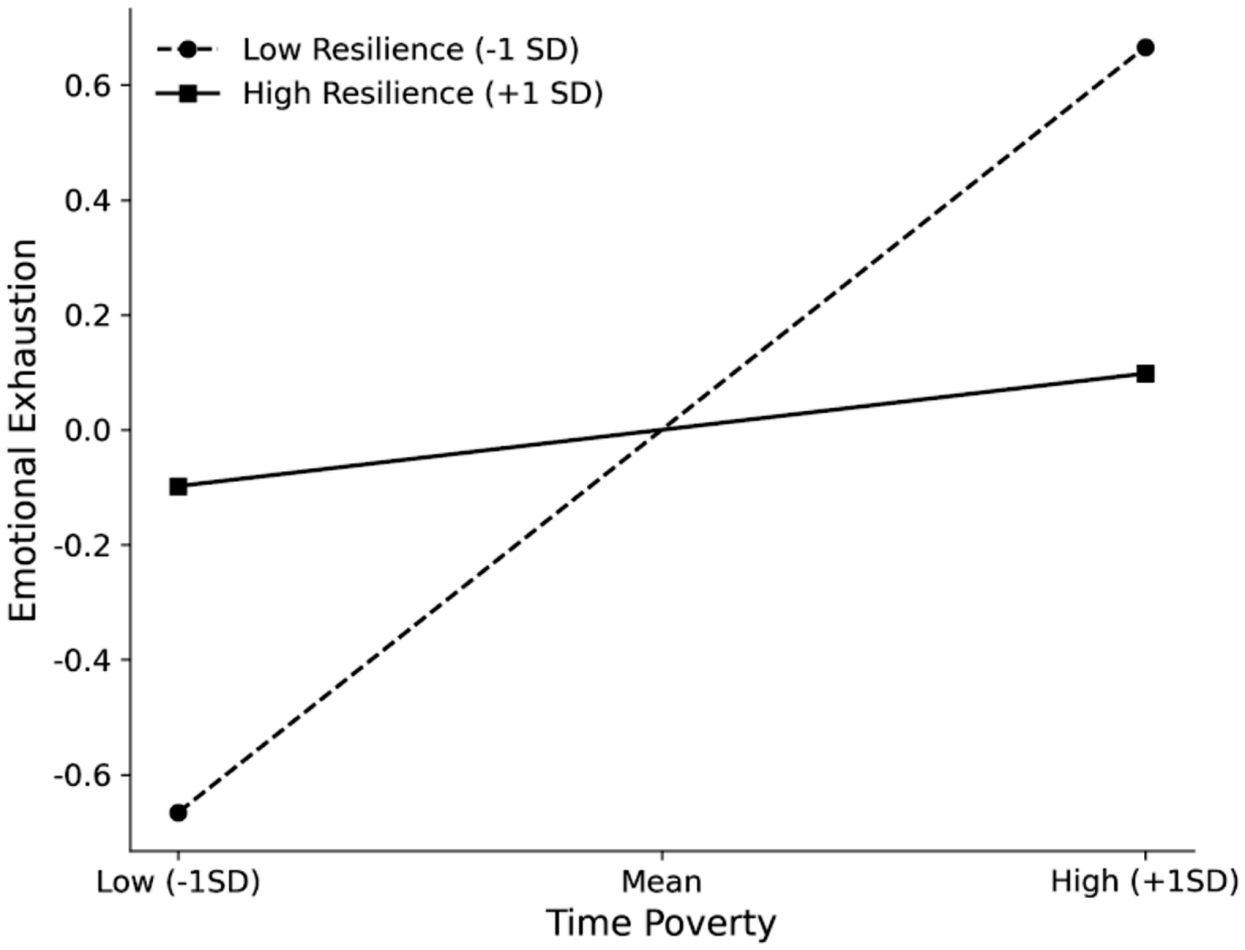
Simple slope analysis of the moderating effect of Psychological Resilience on the relationship between Time Poverty and Emotional Exhaustion. “Low” represents −1 *SD* from the mean; “High” represents +1 *SD* from the mean. The positive association is significantly attenuated for teachers with high resilience.

**Figure 3:**
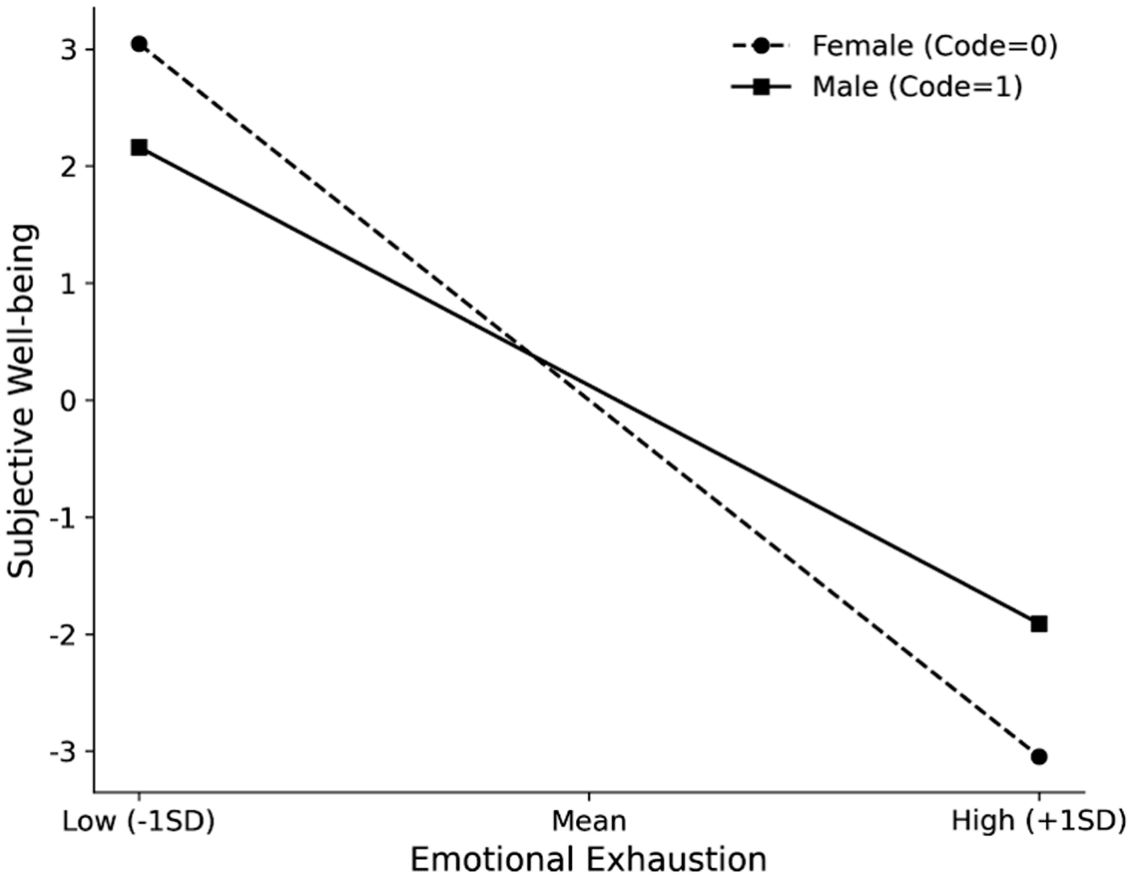
Simple slope analysis of the moderating effect of Gender on the relationship between Emotional Exhaustion and Subjective Well-being. Female = 0, Male = 1. The negative impact of Emotional Exhaustion on Subjective Well-being is stronger for female teachers compared to male teachers.

First, regarding the first stage of the mediation process (Time Poverty → Emotional Exhaustion), the interaction between Time Poverty and Psychological Resilience was significant and negative (*β* = −0.284, *p* < 0.001). This supports Hypothesis 3a. Simple slope analyses (see Figures 2, 3) revealed that the positive association between Time Poverty and Emotional Exhaustion was weaker for teachers with high resilience compared to those with low resilience. However, the moderating effect of Resilience on the second stage (Emotional Exhaustion → Subjective Well-being) was not significant (*β* = 0.002, *p* = 0.935), thus Hypothesis 3b was not supported. Second, regarding the role of Gender, the interaction between Time Poverty and Gender on Emotional Exhaustion was not significant (*β* = −0.038, *p* = 0.617), rejecting Hypothesis 4a. However, the interaction between Emotional Exhaustion and Gender on Subjective Well-being was statistically significant (*β* = 0.138, *p* = 0.002), supporting Hypothesis 4b. Since “Male” was coded as 1 and the interaction coefficient is positive, this indicates that the negative effect of Emotional Exhaustion on Subjective Well-being is attenuated (less negative) for male teachers compared to female teachers. In other words, female teachers experienced a stronger decline in well-being as a result of emotional exhaustion.

## 4 Discussion

Situated within the context of social acceleration and the digital transformation of education, this three-wave longitudinal study examined the mechanisms and boundary conditions linking teacher time poverty to teacher subjective well-being. The results revealed a moderated mediation model that clarifies this complex relationship. Specifically, we found that emotional exhaustion mediated the negative association between teacher time poverty and subjective well-being. Furthermore, this mediation process was moderated at distinct stages by psychological resilience and gender. Psychological resilience functioned as a significant buffer at the first stage of the process, attenuating the positive relationship between time poverty and emotional exhaustion. Gender, in contrast, moderated the second stage of the process: the detrimental effect of emotional exhaustion on subjective well-being was significantly stronger for female teachers than for male teachers.

### 4.1 Time poverty and subjective well-being

Consistent with Hypothesis 1, teacher time poverty was found to be a significant negative predictor of subjective well-being. This finding suggests that the subjective experience of insufficient time, exacerbated by social acceleration, functions as a persistent stressor that impairs teachers’ subjective well-being. The three-wave longitudinal design offers crucial empirical evidence for the causal precedence of time poverty, advancing beyond the correlational limits of cross-sectional research (Batur et al., 2019; Zhu et al., 2025). The results thus establish time poverty as a significant antecedent predicting a decline in teacher well-being, rather than merely a consequence.

This result aligns with the COR theory. As time is essential for acquiring other resources (e.g., income via work, social support via engagement) and for overall well-being (Holmes, 2023; Rosa and Pu, 2022), time poverty constitutes a foundational resource loss under the framework’s “primacy of resource loss” principle. This loss triggers feelings of insecurity and threat, and may provoke defensive withdrawal as teachers attempt to conserve their limited resources (Hobfoll et al., 2018). This coping response, however, prevents them from engaging in necessary resource-replenishing activities. For example, perceived time scarcity may lead teachers to reduce collegial and student-teacher interactions, thereby impairing their school connectedness. Similarly, insufficient time for pedagogical preparation and reflection can undermine their teaching self-efficacy. The erosion of these factors, both of which are core components of well-being (Xie et al., 2024), consequently erodes teachers’ subjective well-being.

### 4.2 The mediating role of emotional exhaustion

Our findings demonstrate that emotional exhaustion mediated the relationship between teacher time poverty and subjective well-being, which supports Hypothesis 2. This suggests that the negative impact of time poverty on teacher subjective well-being also unfolds through the gradual depletion of internal resources. A lack of available time, therefore, appears to function as a chronic stressor that gradually depletes teachers valued emotional and cognitive resources. This process leads to a state of profound exhaustion, which in turn directly impairs their subjective well-being.

This finding aligns closely with the “loss spiral” principle of COR theory (Hobfoll et al., 2018). Within this framework, time is conceptualized as a foundational condition resource. A persistent, perceived deficit of this resource (i.e., time poverty) constitutes an initial resource loss. To compensate for this loss and meet incessant work demands, teachers are forced to over-invest their finite energy resources, such as cognitive effort and emotional labor (Maslach, 2003). Emotional exhaustion is the very state of these energy resources being overextended and depleted, which itself constitutes a significant secondary resource loss. Once teachers enter this state of resource depletion, their capacity to maintain positive collegial relationships or derive a sense of professional accomplishment from their work is severely compromised, directly undermining subjective well-being.

This result also implies that interventions aimed at bolstering teacher well-being may be ineffective if they merely focus on time management skills while ignoring the critical need to replenish and restore teachers’ emotional energy. Once a teacher’s energy resource pool is depleted, they may lack the very psychological capital required to implement new coping strategies.

### 4.3 The moderating roles of resilience and gender

Our moderation analyses identified the distinct roles of psychological resilience (an internal resource) and gender (a sociocultural factor) at different stages of the indirect pathway, clarifying factors that mitigate or exacerbate the negative effects of time poverty.

First, resilience functioned as a “buffer” in the first stage of the mediation pathway. Supporting Hypothesis 3a, high resilience significantly weakened the positive relationship between time poverty and emotional exhaustion. This aligns with the Stress-Buffering Model, which posits that personal resources can attenuate the link between stressors and negative outcomes (Cohen and Wills, 1985). When faced with time poverty, teachers with higher resilience appear more capable of employing adaptive strategies to protect their psychological resources from depletion. This finding extends the known buffering effect of resilience to the context of time poverty. Notably, Hypothesis 3b was not supported; resilience did not significantly buffer the negative impact of emotional exhaustion on subjective well-being. This null finding may suggest that the protective effects of resilience are primarily prophylactic. Once a teacher’s resources are depleted to the point of exhaustion, resilience as a single internal resource may be insufficient to counteract the profound negative impact on well-being.

Second, a key finding is that gender functioned as an “amplifier” in the second stage of the pathway. While Hypothesis 4a was not supported, Hypothesis 4b was, revealing that when experiencing similar levels of emotional exhaustion, female teachers reported a significantly greater decline in subjective well-being than their male counterparts. The lack of support for Hypothesis 4a may suggest that the depleting effect of time poverty is a universal stressor for teachers, regardless of gender. However, the support for Hypothesis 4b reveals a more latent, yet critical, phenomenon: the gender disparity lies not in the susceptibility to exhaustion, but in the subsequent cost to well-being.

This finding can be explained by Social Role Theory (Eagly and Wood, 1999). Women are often socialized with stronger expectations to be “caretakers” and emotional maintainers. Consequently, when female teachers experience emotional exhaustion, they may internalize it not merely as psychological depletion but also as a failure to meet these core social role expectations. The additional burden of this negative self-appraisal could intensify the impact of exhaustion on their well-being to a greater extent than for their male colleagues. This result deepens the understanding of gender differences in teacher well-being by highlighting a specific point of sociocultural vulnerability.

### 4.4 Implications

This study provides empirical support for the application of social acceleration theory within the educational domain. Our results demonstrate that, within the macro-trend of social acceleration, technology-driven educational changes do not automatically enhance teacher well-being. Instead, by creating “time poverty,” they can initiate a detrimental pathway that leads through emotional exhaustion to diminished subjective well-being. This finding offers a crucial empirical counterpoint to techno-optimistic narratives that assume technological progress necessarily alleviates teacher stress. It highlights that structural pressures arising from systemic acceleration, such as time poverty, represent a significant, yet often overlooked, challenge in the digital transformation of education.

Our findings offer several practical implications for mitigating time poverty and improving teacher well-being. The core principle is that interventions must move beyond a sole focus on individual-level enhancement to include systemic changes that foster a supportive, “time-friendly” school environment.

#### 4.4.1 System-level and cultural interventions

School administrators should establish clear digital communication protocols (e.g., a “right to disconnect” policy outside of work hours) and streamline the digital platforms required for daily use to reduce the associated administrative burden. Promoting an efficient meeting culture can also protect blocks of uninterrupted time for teachers to focus on their core pedagogical tasks.

#### 4.4.2 Targeted, evidence-based support

Our moderation analysis provides guidance for more nuanced interventions. First, while resilience-building programs are beneficial, it is crucial to frame them as auxiliary supports that buffer stress, not as panaceas for systemic problems. This avoids the misattribution of a structural issue to the individual. Second, interventions must be designed with gender sensitivity. Our finding that female teachers suffer a greater loss of well-being from emotional exhaustion highlights their unique vulnerability. Therefore, administrators should provide targeted support to mitigate the distinct pressures female teachers face due to societal role expectations. Specific measures could include: 1. Implementing flexible, family-friendly policies that consider teachers’ domestic responsibilities when scheduling non-pedagogical tasks. 2. Fostering peer support networks or mentorship programs for female teachers to share experiences regarding work-life integration. 3. Ensuring the equitable distribution of “invisible work” (e.g., organizing events, extra-curricular duties) to prevent these tasks from disproportionately falling on female teachers.

By implementing such measures, educational institutions can create more equitable working environments that recognize and buffer systemic pressures, thereby supporting the sustainable success of educational digitalization initiatives.

## 5 Limitations and future directions

The present study’s findings should be considered in light of its limitations, which also provide valuable directions for future research.

First, our sample was drawn exclusively from primary and secondary school teachers in China. This context-specificity may limit the generalizability of our findings to other cultural settings or educational systems. Future research should replicate our model in diverse cultural contexts to assess its cross-cultural validity.

Second, all variables were measured using self-report questionnaires, which raises the possibility of common method bias. Although our longitudinal design mitigates some of this concern, future studies would benefit from a multi-method approach. For instance, objective data on workload could be obtained from school records, while daily fluctuations in emotional states could be captured using experience sampling or diary methods. Incorporating physiological indicators of stress, such as cortisol levels, would also provide more comprehensive data. Furthermore, as our measure of time poverty was subjective, future research should integrate objective measures of time use to examine how both perceived and actual time constraints affect well-being.

Finally, while we examined two important moderators, the factors influencing teacher well-being are multifaceted. Future inquiry could expand the model by investigating other potential protective factors (e.g., leadership styles, organizational justice, social support) or risk factors (e.g., role overload). A particularly valuable next step would be to move from observational to experimental research. Designing and testing specific interventions based on our findings, such as resilience-building programs, and evaluating their efficacy in improving teacher well-being via randomized controlled trials, would be a crucial and important avenue for future research.

## Data Availability

The raw data supporting the conclusions of this article will be made available by the authors, without undue reservation.
